# Embryo Cryopreservation in a Patient with Sickle Cell Disease Utilizing Letrozole and Enoxaparin: A Case Report

**DOI:** 10.3390/hematolrep15010011

**Published:** 2023-02-03

**Authors:** Stephanie J. Gunderson, Nina Snowden, Joshua J. Field

**Affiliations:** 1 Department of Obstetrics and Gynecology, Medical College of Wisconsin, Milwaukee, WI 53226, USA; 2 Department of Hematology, Medical College of Wisconsin, Milwaukee, WI 53226, USA; 3 Versiti Blood Center of Wisconsin, Milwaukee, WI 53233, USA

**Keywords:** letrozole, aromatase inhibitor, fertility preservation, sickle cell disease, stem cell transplant, embryo cryopreservation

## Abstract

Purpose: To describe a patient with sickle cell disease, a prothrombotic disorder, who underwent successful embryo cryopreservation for the purposes of fertility preservation prior to hematopoietic stem cell transplant. Methods: To report a successful case of gonadotropin stimulation and embryo cryopreservation using the aromatase inhibitor letrozole to maintain low serum estradiol to minimize thrombotic risk in a patient with sickle cell disease (SCD) and history of retinal artery thrombosis planning hematopoietic stem cell transplant (HSCT). The patient was given letrozole (5 mg daily) as well as prophylactic enoxaparin while undergoing gonadotropin stimulation with an antagonist protocol to preserve fertility prior to HSCT. After the oocyte retrieval, letrozole was continued for one additional week. Results: The patient’s peak serum estradiol concentration was 172 pg/mL during gonadotropin stimulation. Ten mature oocytes were retrieved and a total of 10 blastocysts were cryopreserved. The patient required pain medication and intravenous fluids after oocyte retrieval due to pain but had significant improvement at the scheduled post-operative day one follow-up. No embolic events occurred during stimulation or 6 months thereafter. Conclusion: The utilization of definitive treatment for SCD with stem cell transplant is increasing. We successfully used letrozole to maintain low serum estradiol during gonadotropin stimulation along with prophylactic enoxaparin in a patient with SCD to minimize her risk of thrombosis. This approach will allow patients planning definitive treatment with stem cell transplant the opportunity to preserve their fertility safely.

## 1. Introduction

In the past few years, there have been advances in hematopoietic stem cell transplantation (HSCT) as well as the advent of gene therapy, providing the opportunity to cure more patients with sickle cell disease (SCD). Although promising, both HSCT and gene therapy rely on radiation and/or chemotherapy conditioning regimens to make space in the bone marrow for infused stem cells, often resulting in primary ovarian insufficiency (POI) [1]. Even with the utilization of reduced-intensity conditioning regimens the risk of POI is still significant [1]. Therefore, offering patients fertility preservation options prior to conditioning should be the standard of care [2,3,4].

One of the fertility preservation options available is oocyte/embryo cryopreservation. Traditionally, gonadotropin stimulation required to recruit multi-follicular development is associated with supraphysiological levels of serum estradiol, often 10 times that of physiologic levels [5]. These elevated estradiol levels can be thrombogenic due to estrogen’s effects on procoagulant factors (factor VII, X, and antithrombin III) as well as its stimulatory effect on platelet aggregation [6]. Given that patients with SCD are already at high risk of thrombosis there has been increasing concern regarding the safety of gonadotropin stimulation in patients with SCD [7]. Not only is there concern due to the thrombotic risk to these patients but also the potential of exacerbation of vaso-occlusive pain episodes [7]. Previous case reports of women with SCD undergoing gonadotropin stimulation have utilized prophylactic dose enoxaparin to mitigate the risk of thrombotic events [7,8,9,10]. Aromatase inhibitors, i.e., letrozole, have been utilized to keep estradiol levels within the physiologic range throughout stimulation for patients undergoing ovarian stimulation [11]. Most of the data utilizing letrozole throughout gonadotropin stimulation are in breast cancer patients where the risk of supraphysiologic, sustained estradiol levels could impact their disease [11]. Here we present a successful case of ovarian stimulation for embryo cryopreservation in a woman with SCD utilizing both letrozole as well as enoxaparin to minimize her risk of thrombotic events prior to HSCT.

## 2. Case Report

The patient is a 31-year-old G0 female with SCD. She had been managed with hydroxyurea with infrequent vaso-occlusive pain episodes until, at age 28, she suffered a retinal artery thrombosis, considered equivalent to a stroke, resulting in significant visual impairment. After the stroke, she was maintained on monthly erythrocytapheresis to maintain a hemoglobin S% <30, the established lifelong therapy for secondary stroke. Due to her history of stroke and need for monthly transfusions, she elected to pursue definitive treatment of her SCD with HSCT. Of note, the patient had not experienced any venous thromboembolisms or acute chest syndrome throughout her disease course. Her gynecologic history was significant for menarche at age 13, currently amenorrheic due to Nexplanon implant. Prior to Nexplanon insertion, her menses had been regular, every 28–30 days. She had an updated Papanicolaou smear that was negative for intraepithelial changes and high-risk human papillomavirus. The patient was interested in fertility preservation options prior to her HSCT and was thoroughly counseled on her options.

Once the patient decided to proceed with gonadotropin stimulation, a multidisciplinary team, including specialists in hematology/oncology, reproductive medicine, and anesthesiology, discussed logistics and the best approach for fertility preservation measures. Written consent was obtained from the patient to write this case report.

The patient’s baseline anti-mullerian hormone concentration was 3.19 ng/mL. Antral follicle count measured by transvaginal ultrasound was 17. No evidence of VTE was observed at the start of stimulation. The patient’s pain crises prior to stimulation were well controlled on occasional over-the-counter medications, mainly ibuprofen. The patient underwent an exchange transfusion the day before the start of her gonadotropin stimulation; post transfusion hemoglobin S % was 9.4. 

**Figure 1:**
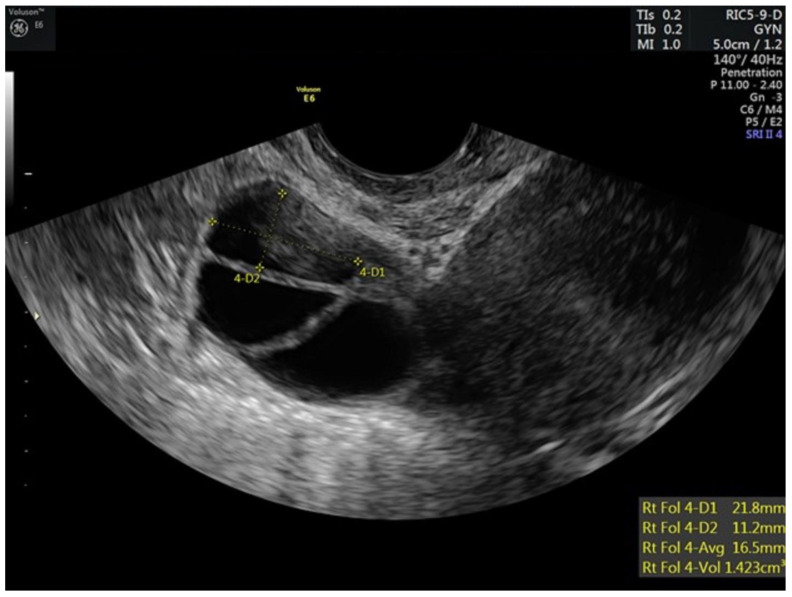
Stimulated right ovary of patient prior to trigger injections.

**Figure 2:**
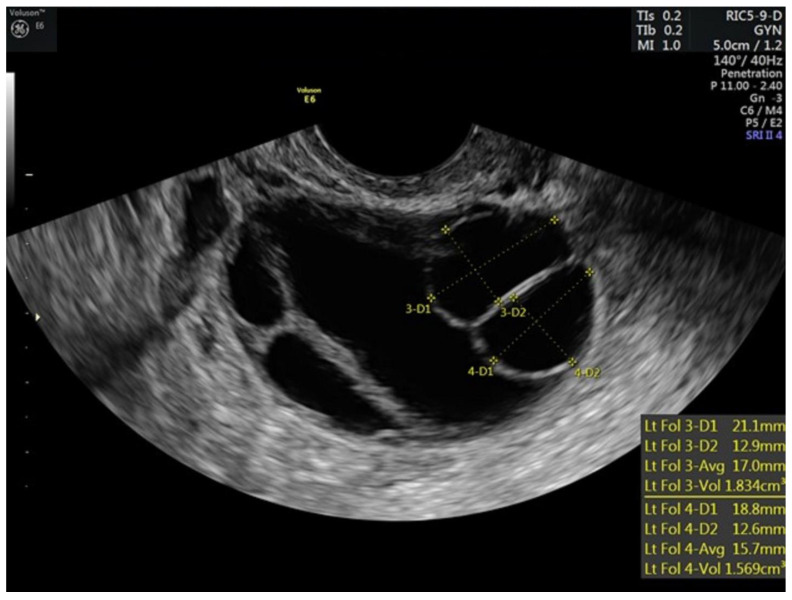
Stimulated left ovary of patient prior to trigger injections.

The patient began gonadotropin stimulation in July of 2021. She began oral letrozole and prophylactic enoxaparin, at 40 mg daily, 1 day prior to stimulation. She was started randomly as she had a hormonal intrauterine device in place. At baseline, her estradiol concentration was 35.5 pg/mL and her transvaginal ultrasound antral follicle count was 21. After 3 days of stimulation, the patient’s estradiol concentration was 74.1 pg/mL and follicular growth was noted. Transvaginal ultrasounds and estradiol levels were assessed every two days to assure progression of follicular maturation (Table 1). She received a total of 1075 IU recombinant follicle stimulating hormone (rFSH) and 750 IU human menopausal gonadotropin (HMG) over 11 days. When the lead follicle was >12 mm a gonadotropin releasing hormone antagonist was started to prevent premature ovulation, which was utilized on stimulation days 6–10. Her peak estradiol concentration was 172 pg/mL. When there were two lead follicles ≥18 mm an injection of 1500 units of human chorionic gonadotropin (hCG) and 40 units of gonadotropin-releasing hormone agonist (GnRH-agonist) were given to trigger follicular maturation and resumption of meiosis (Figure 1 and Figure 2). Enoxaparin was given on the day of the final maturation trigger and then held until post operative day #1. Her luteinizing hormone concentration on the day of final maturation trigger injections was 0.6 mIU/mL Oocytes were retrieved under conscious sedation 34 h later. Of the 10 oocytes retrieved, 10 were mature, all 10 mature oocytes were successfully fertilized with intracytoplasmic sperm injection (ICSI) and 10 blastocysts were created (six A quality and four B quality). Semen parameters on the day of oocyte retrieval were as follows: sperm concentration = 60 million/mL, total motility = 33%, and progressive motility = 28%.

Immediately after the procedure, the patient required intravenous (IV) and oral opioids to reduce her pain. The patient was discharged from the reproductive medicine facility to the sickle cell clinic for IV fluids.

At her 1-day scheduled follow-up, she reported her pain as minimal. A transvaginal ultrasound revealed trace-free fluid in the cul de sac, her right ovary was 5.2 × 4.7 × 4.1 cm, and her left ovary was 5.4 × 5.2 × 4.3 cm. Her physical exam was consistent with a soft abdomen, and mild tenderness to palpation over the right lower quadrant. No further follow-up was needed.

The patient underwent a successful HSCT in September of 2021, approximately 2 months after her gonadotropin stimulation. There was no follow-up anti-müllerian hormone level after HSCT. At her most recent follow-up appointment, she was doing well, without evidence of graft versus host disease with full engraftment. Her hemoglobin at the time of follow-up was 12.5 g/dL.

## 3. Discussion

Curative therapies, both HSCT and gene therapy, are likely to become more common in patients with SCD in the coming years. Advances in HSCT in SCD, including reduced-intensity conditioning regimens and alternative donor strategies, will allow more patients the opportunity for transplantation. Gene therapy trials in SCD, of which there are seven currently open, offer additional opportunities for curative therapy and will likely change the treatment paradigm for patients with SCD. The risk of POI associated with the conditioning regimens prior to HSCT and gene therapy is often overlooked and, as a result, so are offering fertility preservation services [3,4].

To our knowledge, this is the first case report to successfully utilize letrozole for estradiol suppression during gonadotropin stimulation in a patient with SCD undergoing embryo cryopreservation for fertility preservation. Patients with sickle cell disease, due to the nature of the disease, have a high risk of VTE and pain crises. It is well known that estradiol affects the blood clotting mechanisms by increasing procoagulant factors (factor VII, X, and antithrombin II) as well as stimulating platelet aggregation [6]. During gonadotropin stimulation, estradiol levels commonly reach supraphysiologic levels, therefore there is concern that patients undergoing stimulation may be at increased risk of VTE [5]. Letrozole, utilized to keep estradiol levels physiologic, has been used effectively in patients with estrogen receptor-positive breast cancers undergoing gonadotropin stimulation [12]. Letrozole does not appear to reduce mature oocyte yield [8] and generally is a well-tolerated oral medication. To this end, utilization of letrozole in patients with pro-coagulant disorders, like SCD, would theoretically lessen the risk of VTE during gonadotropin stimulation. To further minimize estrogen rise, a GnRH agonist can be used instead of hCG to trigger oocyte maturation [13].

Determining the appropriate trigger for final maturation of oocytes is important, especially in a patient undergoing gonadotropin stimulation to preserve their fertility. Often these patients have the opportunity to bank eggs or embryos *once*; therefore, maximizing their response and oocyte maturation is key. One of the known risks of gonadotropin stimulation is ovarian hyperstimulation syndrome (OHSS) [14]. The physiology of OHSS includes increased vascular permeability because of rising levels of vascular endothelial growth factor (VEGF) [14]. VEGF is thought to rise in response to high levels of hCG either through pregnancy or due to utilization of hCG triggers for final maturation of oocytes [10]. Therefore, minimizing doses of hCG at the time of trigger of final oocyte maturation is important for those at risk of VTE. In our patient, we utilized a dual trigger with a GnRH agonist and a small amount of hCG. Dual triggers have been shown to minimize the risk of OHSS without impacting mature oocyte yield [15]. Another alternative is to utilize a GnRh-agonist trigger without hCG for final maturation of oocytes. Both dual trigger and GnRh- agonist triggers have been shown to minimize the risk of OHSS without impacting final oocyte maturation [15]. We believe that *either* of these triggers of final oocyte maturation would be appropriate in SCD patients as both have been shown to mitigate the risk of ovarian hyperstimulation [15]. In our clinical practice, we tend to utilize dual triggers rather than GnRH-agonist triggers alone to minimize patients having to return for blood work to assure a successful endogenous response to a GnRH-agonist trigger.

A few case reports have utilized enoxaparin in sickle cell patients undergoing gonadotropin stimulation for fertility preservation [7,8,9,10]. In comparing our case to other case reports of patients undergoing fertility preservation procedures, our patient had similar oocyte yields with excellent fertilization and blastulation rates, offering reassurance that letrozole does not appear to affect oocyte maturity or embryo yield [12]. We recognize that it is not typical for all mature oocytes retrieved to fertilize and develop into good-quality blastocysts eligible for cryopreservation. It is well established that ICSI is associated with 70% fertilization rates [16]. Rates of blastulation are dependent on infertility diagnosis, oocytes quality, and culture conditions and have been reported to range between 39 and 53% [17,18,19]. Our patient exhibited excellent fertilization and blastulation rates allowing for multiple attempts to achieve future pregnancies.

Some difficulties did arise during this case. IV access was limited which led to limited IV fluid hydration throughout the egg retrieval and ultimately a pain crisis. Pain crises have been described in case reports of sickle cell patients undergoing gonadotropin stimulation for fertility preservation, so we were prepared for this. We were able to quickly communicate with her hematology team and transfer her to the infusion center a few hours after her procedure for proper IV hydration and pain management. The quick transition of care from our outpatient IVF center to a hospital-based infusion center highlights the importance of high levels of care coordination for patients with significant co-morbid conditions undergoing gonadotropin stimulation for fertility preservation. The authors acknowledge that this is a single patient case report making generalizability limited. As definitive management for SCD increases, care coordination and an understanding of the unique risks these patients face while undergoing gonadotropin stimulation will be important in keeping patients safe. Larger case series are needed to determine if enoxaparin anticoagulation is needed during gonadotropin stimulation in patients with sickle cell disease or if letrozole, for estradiol suppression, is sufficient in minimizing thrombotic risk in this population while undergoing gonadotropin stimulation.

## 4. Conclusions

As definitive treatment for SCD increases, there will likely be an associated rise of patients interested in fertility preservation procedures prior to HSCT. Maintaining low estradiol concentrations with letrozole to minimize VTE events while potentially avoiding anticoagulation with enoxaparin in patients with SCD during gonadotropin stimulation will allow patients planning definitive treatment with HSCT the opportunity to preserve their fertility safely.

## Figures and Tables

**Table 1:** Stimulation Summary.

Cycle Day	Medication Dosing							Serum Testing		Right Ovarian Follicles (mm)	Left Ovarian Follicles (mm)	Endometrial Thickness (mm)
	Letrozole (mg)	Enoxaparin (mg)	rFSH (IU)	HMG (IU)	GnRH Antagonist (mcg)	GnRH Agonist(IU)	hCG(IU)	Estradiol (pg/dL)	LH (IU/L)			
0	5											
1–3	5	40	100	75				35.5		12 < 10	9 < 10	3.3
4–6	5	40	100	75				74.1		7 < 10	9 < 10	4.1
7	5	40	100	75	250			84		12,11,10,9,9,7	12,12,11,10,9,8	4.7
8–9	5	40	100	75	250			116		15,14,13,11,10,9	16,14,13,13,12,7	5.8
10	5	40	100	75	250			172	0.6	19,17,17,13,12,12,9,6	18,17,16,16,13,4	5.3
11	5	40	175	0		40	1500					

## Data Availability

Not applicable.
